# Patient Perspectives of Living with Coeliac Disease and Accessing Dietetic Services in Rural Australia: A Qualitative Study

**DOI:** 10.3390/nu13062074

**Published:** 2021-06-17

**Authors:** Rachelle Lee, Elesa T. Crowley, Surinder K. Baines, Susan Heaney, Leanne J. Brown

**Affiliations:** 1 School of Health Sciences, College of Health, Medicine and Wellbeing, University of Newcastle, Callaghan, NSW 2308, Australia; rachelle.lee@health.nsw.gov.au (R.L.); surinder.baines@newcastle.edu.au (S.K.B.); susan.heaney@newcastle.edu.au (S.H.); 2 Dubbo Health Service, Western NSW Local Health District, Dubbo, NSW 2830, Australia; 3 Department of Rural Health, College of Health Medicine and Wellbeing, University of Newcastle, Tamworth, NSW 2340, Australia; elesa.crowley@health.nsw.gov.au; 4 Tamworth Rural Referral Hospital, Hunter New England Local Health District, Tamworth, NSW 2340, Australia

**Keywords:** coeliac disease, dietitian, rural health services

## Abstract

Adapting to living with coeliac disease requires individuals to learn about and follow a strict gluten-free diet. Utilising a qualitative inductive approach, this study aimed to explore the perspectives of adults diagnosed with coeliac disease who have accessed dietetic services in a rural outpatient setting. A purposive sample of adults with coeliac disease who had accessed dietetic services from two rural dietetic outpatient clinics were recruited. Semi-structured interviews were conducted by telephone. Data were thematically analysed. Six participants were recruited and interviewed. Three key themes emerged: (i) optimising individualised support and services, (ii) adapting to a gluten-free diet in a rural context, and (iii) managing a gluten-free diet within the context of interpersonal relationships. Key issues identified in the rural context were access to specialist services and the increased cost of gluten-free food in more remote areas. The findings of this study have highlighted the difficulties associated with coeliac disease management and how dietetic consultation has the potential to influence confidence in management and improve lifestyle outcomes. Further qualitative research is required to expand on the findings of this study and inform future dietetic practice that meets the expectations and individual needs of people with coeliac disease in rural settings.

## 1. Introduction

Coeliac disease is often misdiagnosed or delayed in diagnosis, with dietary restrictions that may not be suitable or well understood. Managing and adapting to a chronic disease with strict dietary requirements is a challenge. Having to do so in a rural context may be further challenged due to a disparity of access to health care [1] and increased costs of food and availability issues [2]. The needs of the person requiring a specialised diet may compete with the food needs of other members of the household as well as expectations in social interactions [3].

Current treatment for coeliac disease includes strict life-long adherence to a gluten-free diet, [4] requiring complete avoidance of gluten-containing grains (wheat, barley, and rye) and their by-products [5]. As well as avoiding gluten, a balanced diet with adequate vitamins, fiber, and calcium is essential [6]. This permanent dietary restriction has a major impact on the nutritional adequacy of the diet and the quality of life of both the person with coeliac disease and those around them [7,8]. Factors that predict or influence long-term health outcomes include genetics, environmental factors, ongoing inflammation of the small intestine, and nutritional deficiencies [9]. Common problems associated with adjusting to a gluten-free diet include a lengthy education process and the identification of gluten-free foods that are affordable and enjoyable [8,10,11,12]. Eating out of the home and socialisation may also become more difficult under the constraints of the gluten-free diet, and feelings of social isolation, worry, and neglect are also commonly reported [11,12].

It has been suggested that those with coeliac disease require encouragement, motivation, and support from a collaborative medical and dietetic team to ensure adherence to the gluten-free diet and subsequent progressive treatment outcomes [13]. Improvements in practitioners’ abilities to educate about coeliac disease has been linked to potential for improved adherence to a GF diet [14]. The British Society of Gastroenterology recommends that individuals with coeliac disease should attend a dietetic consultation and counselling session upon diagnosis, at three and six-months post-diagnosis, and then be reviewed annually by both a dietitian and the treating physician [13,15]. Despite these recommendations, some literature indicates that the availability and use of dietetic services in the management of coeliac disease is less than adequate [15,16].

Coeliac disease is a complex condition that can be difficult to manage [17], particularly in a rural or regional setting, where access to a wide variety of gluten-free foods and specialised health care services is limited. Current research has highlighted the importance of dietetic consultation post-coeliac disease diagnosis [13]; however, a number of studies indicate that individuals with coeliac disease are not being followed up adequately [10,16]. There has been limited exploration of patient expectations and satisfaction with dietitian consultations.

Due to the complexity of the gluten-free diet, specialist advice and dietary education are important in preventing inadvertent gluten consumption and persistence of symptoms [7,18]. Few dietitians specialise in coeliac disease management [5,15,16], and rural-based dietitians may be less experienced. Rural-based dietitians may have a generalist role with a broad case-load and limited opportunities for specialisation and consequently, less experience with the dietary management of coeliac disease [19,20].

Current evidence investigating coeliac disease from the qualitative perspective [8,11,12,17,21,22] has focused on patients’ experiences [11,12], perspectives of close relatives [8], diagnosis and realities of living with coeliac disease [17], and motives for adherence to a gluten-free diet [21]. Qualitative explorations specific to health professional care have explored experiences of dietetic consultations [22,23]. Further research is required to develop a greater awareness of the condition and its impact, especially in terms of access to dietetic services in different settings [8,12,17]. This study aimed to explore patient perspectives of use of dietetic services and their ongoing management of coeliac disease in a rural setting.

## 2. Materials and Methods

Study design: This qualitative study used a general inductive approach [24] to explore the perspectives of adults with coeliac disease. Patients who attended an outpatient dietetic clinic in a regional or rural setting of a Local Health District (LHD) regarding dietary advice for coeliac disease (between July 2015 and May 2019) were purposefully selected. The LHD sites were located in Modified Monash Model locations [25] classified as MM3 (large rural town) and MM4 (medium rural town). From two health care settings and a total of 19 eligible participants, six agreed to be interviewed. Subjects were eligible if they had been clinically diagnosed with coeliac disease, attended a dietetic consult within the past five years, and if they were able to participate in a telephone interview. Those under the age of 18 years at the time of consult were excluded.

Ethics: Ethics approval was obtained through the relevant NSW Health Local Health District Human Research Ethics Committee (15/05/20/5.01) and the University of Newcastle Human Research Ethics Committee (H-2015-0165). Anonymity of potential participants was maintained by having a member of hospital administration staff manage the mailed invitations. Consent was sought from all participants for both the data they provided to be utilised for research and their interview to be audio recorded. Analysis of results was undertaken by researchers who were not known to the participants (RL and LB).

Recruitment: An information statement explaining the inclusion and exclusion criteria, expectations of participants, rights of participation, and privacy were mailed out to all eligible participants along with a consent form. Those who consented were contacted by telephone for an interview after they returned the consent form via a pre-paid envelope.

Development of the data collection tool. A review of the literature informed the development of the semi-structured interview protocol. A search of databases Medline, CINAHL, and PubMed enabled the identification of common themes around issues in the management of coeliac disease and revealed gaps in current knowledge. These gaps were addressed through the development of interview questions about their use of dietetic services, health outcomes, symptom management, and quality of life in an attempt to improve the knowledge base and provide a more holistic understanding of the perspectives of individuals with coeliac disease.

Data collection: Semi-structured interviews recorded and ranged from 15 to 45 min in duration (Supplementary File). With verbal confirmation of consent, the interviews were audio recorded and transcribed verbatim. A series of probes and prompts were used during the interviews. Transcripts were checked for accuracy against the original recording and assigned a non-identifiable code. Interviewer field notes were completed for each interview by the interviewer. Transcripts were checked against the audio recording for accuracy. Participants were provided the opportunity for member checking, to ensure the information derived from the transcription was valid. Transcripts were read and coding developed with the assistance of NVivo 10 (QSR International Pty Ltd. 2015, Doncaster, Victoria, Australia). Categories and themes were developed and revised (RL) with another a second experienced researcher (LB). Themes were developed using a general inductive approach [26]. Excerpts from the interviews were edited for clarity and anonymity. The Standards for Reporting Qualitative Research checklist was utilised when reporting this research [24].

The size of the sample was largely determined by the availability of respondents who were willing to participate in an interview. A sample of six participants was deemed appropriate because of the exploratory nature of this research and the focus on identifying the experiences of those in a rural area. This study did not attempt to examine a representative sample to provide a comprehensive understanding of all possible experiences, but rather to explore issues within a rural context with a purposeful sample from a rural area with access to a specific dietetic service.

## 3. Results

Six people were interviewed, three male and three female, with an age range from 38 to 77 years; number of years since diagnosis ranged from <1 year to 10 years. Participants resided in MM3 (large rural towns) or MM5 locations (small rural towns). A summary of participant (P1–P6) demographics, their symptoms at diagnosis, and their dietetic management is provided in Table 1. Half of the participants attended more than one appointment with a dietitian, with one seeking follow-up appointments due to later tests showing ongoing inflammation of the small bowel.

Three key themes were identified through analysis of the data. These related to (i) optimising individualised support and services, (ii) adapting to a gluten-free diet in a rural context, and (iii) managing coeliac disease within the context of interpersonal relationships. Table 2 provides a summary of the themes and sub-themes with supportive quotes.

### 3.1. Optimising Individualised Support and Services

#### 3.1.1. Provision of Support and Services Relevant to Patient Needs

Experiences with dietetic consultations were varied. Most participants found their consultation helpful and were positive about the role of the dietitian in coeliac disease management. One participant who described a ‘two-way’ process was positive about the experience and considered it worthwhile, expressing contentment with the dietitian in tailoring the consultation to his needs, and this was reflected in his willingness to return for necessary follow-up consultations. Another participant who described that he was ‘on the right track’ with his diet was ‘pretty happy’ with his experience.

Contrary to this, a participant was less satisfied with her experience, suggesting that the education she received did not align with her expectations, nor was it tailored to her needs, suggesting that the approach taken was not individualised and that her level of understanding was already high. Those with limited access to consult with a dietitian, due to limited outreach services, relied on other sources of information. One participant felt they mainly obtained information from books and that access to a dietitian was difficult due to the lack of a local service.

#### 3.1.2. Meeting the Expectations of the Patient

Satisfaction with the dietetic consultation was linked to initial expectations of what the service was going to offer. P1 had specific expectations around the type of information that was to be provided and expressed that ‘*I would expect my dietitian to point [that] out to me*’. Other participants reported a more positive experience and indicated that their expectations had been met, indicating that the dietitian ‘*gave me all the tools and skills the skills*’. Expectations were varied, with some expectations going beyond advice about food.

#### 3.1.3. Consistency in Communication and Coordination of Care

The unmet expectations of P1 were linked with inconsistent communication within the health-care team, with the implication of a lack of communication between the doctor and the dietitian. ‘*I think there needs to be a lot more conversing, communication between the doctors and the dietitians…’ (P1).* This contributed to confusion around the roles of these health professionals and lack of confidence in the health-care team. Other participants identified ways in which health professionals could support people with coeliac disease by understanding and supporting them to make their own choices. P6 stating that the dietitian ‘…*helping you to move forward… as the GP did as well, like we all have choices….’*

#### 3.1.4. Improving Services and Resources

Two participants (P1 and P3) provided suggestions to improve support and services offered to coeliac disease patients. These included increasing access to educational resources and other services to support those managing a gluten-free diet, for example a social media page. It was also suggested that management would become easier and more satisfying with education and skills around gluten-free cooking. Other participants mentioned other resources they used and found helpful such as the Coeliac Society website and gluten-free cookbooks. Resources related to gluten-free cooking were highlighted *‘…another book on gluten-free cooking… that I use a lot…’* (P6) as very useful.

### 3.2. Adapting to a Gluten-Free Diet in a Rural Context

#### 3.2.1. Confidence Around Managing Ones’ Coeliac Disease

All participants indicated that confidence in management of their coeliac disease developed over time and with increasing education. Despite a sense of self-managing their coeliac disease and expressing no desire to return for follow-up consultations with a dietitian, P1, P2, and P4 expressed a lack of confidence in their ability to determine if a food was gluten-free or not. These participants limited the variety of their diet and relied heavily on familiar and packaged foods labelled gluten-free: *‘If it’s got ‘gluten-free’ on it, well, I buy it’* (P4). Another participant indicated further awareness of this, suggesting the importance of education around gluten-free products for greater dietary enjoyment and flexibility. ‘*You miss out on the pleasure of tasting something simply because you don’t…. recognise that it is within the bounds of what you can… consume’* (P3).

#### 3.2.2. Adapting to and Maintenance of the Gluten-Free Diet

All participants noted improvements in their physical health after commencing the gluten-free diet; however, most reported situations where the inadvertent consumption of gluten caused ongoing symptoms. Despite positive physical outcomes, frustration was expressed at the length of time taken to adjust to and feel better on the gluten-free diet. ‘*It shouldn’t be that hard I don’t think’* (P1).

Adaptations to social situations were described as more difficult to control and could lead to inadvertent gluten consumption. ‘… *I might have some gluten in my diet purely because I might be in a social situation…’* (P6). A participant also reported on the importance of developing a new skill of reading food labels for better management of their condition. *I’ve never read a label before, now I’ve got to’* (P2).

Adaptation to a gluten-free diet and management was made more difficult for some participants due to the higher cost and lower quality of gluten-free foods, with the cost reportedly ‘*usually double’ or ‘three times the price’* that of a gluten containing option. Staple foods such as bread and baked products were identified as particularly expensive and difficult to access in some rural areas. The higher cost of gluten-free foods necessitated the development of skills in managing a low income and *learning ‘how to shop within [a] budget* (P6). Most participants expressed their dissatisfaction with the majority of gluten-free products, particularly staple foods such as bread, ‘*I get the worst bread’,* and pastries: *‘I get a second-rate meat pie’* (P2).

#### 3.2.3. Food as a Major Aspect of Life

Most participants communicated difficulties in managing their coeliac disease with food being a major aspect of everyday life. P1 and P2 spoke of the effect of the gluten-free diet on their ability to attend social events: *‘I find eating out is just awful, I dread it sometimes’* (P1). Meanwhile, P3 reported a decreased enjoyment in life secondary to the elimination of gluten-free foods. Another participant found it easier to manage at home where *‘everything is gluten-free’* (P5).

A participant (P6) did not find the change particularly difficult due to a *‘basic sort of diet’* she followed at home prior to diagnosis. Despite this, she still found she needed to look for suitable alternatives. The same participant expressed that the challenge was with the impact of the dietary change on her weight ‘*because a lot of the products had extra sugar.’*(P6)

While access to food was not always an issue in the location of residence *‘where I live, there’s a good range of gluten-free products’* (P6), others had more difficulties. Living in a small rural town was linked to limited gluten-free options and required travel to larger centres to access varied options. Some found travel difficult particularly in more remote areas where the food was more expensive and there are fewer fresh food options that are available.

#### 3.2.4. Resignation to the Diet and Lifestyle Changes

The participants responded differently to the chronic nature of coeliac disease. One was forthcoming with emotions around the long-term nature of the disease, acknowledging feelings of sadness. ‘*It’s a bit depressing, yeah, definitely’* (P1).

Some participants (P2, P3, P6) expressed a greater acceptance of the condition; they implied that withdrawal of emotional involvement was how they coped with coeliac disease. Another participant talked about the challenges of adapting her shopping and cooking and how she became ‘accustomed to’ the dietary changes over time, *‘…in time, you manage… you get accustomed to it and you can get some pretty good results’* (P6).

Being newly diagnosed, P4 had less experience in long-term coeliac disease management but had a positive outlook for the future: *‘I’m confident that I’ll be able to manage the thing… time will tell’* (P4). Another participant with a long-standing diagnosis said, *‘I just accept it… It’s just what I need to do and I just do it’* (P6).

### 3.3. Managing Coeliac Disease within the Context of Interpersonal Relationships

#### 3.3.1. The Role of Others

The way ‘others’ reacted to the demands of the gluten-free diet was raised by all participants. It was implied that these reactions affected the self-esteem and confidence of participants, particularly within the social context. This contributed to a decline in social activity and a reluctance to attend social events where food is a major component.

The embarrassment of others when unable to provide for those with special dietary needs was also a key issue, reinforcing the onerous impact of the gluten-free diet and its contributing to their social withdrawal. With P1 expressing that ’*I just felt like the biggest pain in the neck’*.

Difficulties in getting some family members to understand dietary needs led to participants opting to choose to not make it ‘too hard for them’. Whereas a male participant had a more positive experience with socialising, which was fostered through long-term relationships with a stable social group who were aware of his special dietary requirements and willing to assist.

Another male participant approached the involvement of others from a different perspective, suggesting their acceptance or otherwise should not affect his management. ‘*I’ve got to look after number one.’* (P4) In contrast, another participant (female) was more apologetic and conflicted by the imposition of her diet on others. *‘It just becomes too hard for them and I don’t want that to be the case’* (P6).

The social emergence of the gluten-free diet was raised in both a positive and negative light. P1 spoke of the stigma around the gluten-free diet and the difficulties associated with communicating the seriousness of the condition. *‘I’ve had café [staff] actually just like roll their eyes at me…’* (P1). Meanwhile, others suggested that the popularity of the gluten-free diet has facilitated the availability of new food products and greater dietary variety for people with coeliac disease.

#### 3.3.2. Management of the Diet and Disease within the Family Unit

Ideas around management of the diet within the family unit was raised by two participants. P3 described living in a household that was ‘completely gluten-free’, and he expressed empathy for his wife in missing out on ‘all the good foods’ (P3). P1 spoke of the difficulties associated with raising and feeding children in a partially gluten-free household, and some debated the decision of whether or not to eliminate gluten from the household all together. *‘I don’t know whether to go fully gluten-free in this house’* (P1). Others commented that there were no issues when eating with family members in various locations with family members opting to choose to eat gluten-free at home and in their presence.

#### 3.3.3. Responsibility for the Diet and Disease

Two participants (P1 and P2) indicated that they were autonomous in managing their gluten-free diet and had no expectations for those around them to take on this responsibility.

The challenge of juggling social situations that impact on taking responsibility for one’s own food intake and the health implications was highlighted by P6. Others made the decision to not put themselves in a position to have gastrointestinal symptoms due to relying on others to understand the requirements of a gluten-free diet

The role of the partner or spouse in the management of coeliac disease was also raised with some partners being engaged and involved in supporting their partner with their gluten-free dietary requirements and others less engaged. P1 expressed the difficulties in educating her husband about gluten-free foods. In contrast to this, P3 described being completely reliant on his wife for his food and dietary management. This participant expressed his gratitude for his wife’s involvement and indicated that he should learn to take more control of his coeliac disease and demonstrated an awareness of how difficult self-management would be.

## 4. Discussion

This study has been the first of its type to provide insights into the perspectives of individuals with coeliac disease who have accessed dietetic services in a rural outpatient setting. This research has described the difficulties associated with living with coeliac disease and the impact this has on an individual’s social interactions and relationships. The findings have revealed some strengths and inconsistencies in the dietetic management of coeliac disease in a rural setting. The explorative findings from this study can inform future dietetic practice in coeliac disease management in rural settings.

In this study, some participants experienced lengthy delays to diagnosis, which is common for patients with coeliac disease [7,11,16]. Misdiagnoses are also common [17], which can be traumatising and frustrating for many people with coeliac disease [17]. Post-diagnosis, participants had variable experiences and levels of expressed satisfaction with the dietetic services provided. The dissatisfaction reported by one participant appeared to originate from a dietetic consultation that failed to meet her expectations and inadequate communication within the health-care team. This was also identified in another qualitative study based in Victoria, Australia [17], where 10 women (aged 31 to 60 years) with coeliac disease who were members of a state-based coeliac society were interviewed. Others have reported on the importance of considering patient expectations [22] in general dietetics consultations. These concepts have not previously been explored in the management of coeliac disease. Our study also suggests the need for greater interdisciplinary communication for improved patient outcomes. This also raises issues of understanding health care roles delineation (i.e., who covers what issues) and dietary versus non-dietary topics of discussion. This may be particularly important in rural areas where clinicians may have less opportunity to specialise or gain regular experience in particular fields of practice.

Research has found that people with coeliac disease expect to be provided with information specific to their lifestyle when attending dietitian consultations [23]. The three participants who returned for follow-up had positive reports about their dietetic consultations; they indicated that the interventions were well suited to their lifestyle and that the provision of skills and knowledge was adequate. Despite occasional difficulties in management, these participants felt they required no further follow-up. This was secondary to issues including rurality, which made access to dietetic services more difficult for one participant. These findings have not been previously explored through qualitative research.

All participants in this study reported improvements in physical health after commencement of the gluten-free diet. Other evidence demonstrates holistic improvements in the physical and mental health of those with coeliac disease after gluten-free diet commencement [17]. Confidence in coeliac disease management appears to develop over time as knowledge and insight into the requirements of the gluten-free diet increase. In the early years after coeliac disease diagnosis, one of the greatest difficulties experienced by patients is determining which foods are safe to eat. The accidental consumption of gluten containing foods is also common [12], which is a major source of dissatisfaction and self-reported poorer treatment outcomes among those with coeliac disease in this study. Decreased enjoyment in food is another key factor affecting the quality of life of patients with coeliac disease found in this and other studies [11,12,17]. In agreement with other research [17], the need to be compliant with the gluten-free diet and limited variety of gluten-free foods, particularly in rural areas, was a source of constant disappointment for most participants in this study. Interestingly, male participants appeared to be more accepting and less apologetic about their specialised dietary needs, while female participants tended to be apologetic or accommodating to fit in with their family members and social group.

The popularity of the gluten-free diet to treat ailments apart from coeliac disease is rising [17], which has also led to an increase in the availability and variety of gluten-free food products. King et al. [27] refer to this as the “double edged sword” of greater availability of gluten-free options but also the risk of contamination due to the undermining of the seriousness of the strict dietary needs of people with coeliac disease.

The sense of self-worth for patients with coeliac disease appears to be shaped by the reactions of those who play a meaningful role in their lives. Participants in this and other studies report feeling like a burden on those around them [8,12,17]. This study indicates that people with coeliac disease seem more comfortable with those who understand and are willing to cater for their dietary requirements, which another study suggests, increases their willingness to socialise [17].

The one female participant of this study reported an adverse emotional reaction to having coeliac disease. This issue has also been explored by other researchers [28], who suggest women find the disease more socially confining and tend to struggle with feelings of loss of control [29]. Furthermore, management of the gluten-free diet seems easier when responsibility is shared with a spouse or significant other, as reported by some participants in this study. Taylor et al. [17] have also reported that the burden of the diet and lifestyle were lessened when the load was shared within the relationship [17]. Access to quality resources such as those provided by the Coeliac Society were identified by participants as useful. Membership of a coeliac association has been linked to improvements in adherence to a gluten-free diet in a systematic review [14].

This purposeful sample provides insights into the experiences of rural-based people with coeliac disease accessing a free dietetic service. While a lack of information about education level and a limited age range of participants (38 to 77) in this study limits the generalisability of the findings, the purpose of the study was be exploratory and not to make the findings generalisable. The ages of the participants in this study may reflect the nature of those attending a free dietitian clinic at a rural LHD. Private practice dietetic services may be an alternative source of service provision for those who have the ability to pay a full consultation fee or who have private health cover. Although our sample size was considered sufficient for this exploratory study, further research with a range of diverse participants may provide additional insights. Further research may be required to explore the experiences of a diverse range of people with coeliac disease.

Due to the qualitative nature of this research, any associations made are open for interpretation and therefore subject to a degree of researcher bias. To minimise this, coding was cross-checked by at least two researchers. It could be considered that there may be a non-responder bias in that those less satisfied with the service did not respond to the invitation to participate. Despite this, there was a diverse range of experiences, and opinions were found in this pragmatic study with the sample of participants, which represent one-third of all who were eligible in the pragmatic timeframe of this study. Finally, this study did not explore the experiences and those living in a rural area who had not consulted a dietitian or those who may have sought consultation from a private practice dietitian. Individuals who did not access dietetic services or who sought private practice services may have other diverse experiences that are yet to be explored.

Findings from the current study suggest a need for a consistent but individualised approach to coeliac disease management, as the number of dietetic consultations was variable among the participants and participant needs were varied. Individualised counselling based on the expressed needs and existing knowledge of patients was also identified as important for patient engagement. Finally, greater interdisciplinary communication and a consistent and comprehensive nutrition assessment is needed to gain a deeper understanding of the priorities and expectations of patients; this could lead to optimal patient satisfaction.

## 5. Conclusions

The findings of this study highlight that adapting to a gluten-free diet to manage coeliac disease can be challenging for some people, and this can be exacerbated by living in a rural context. The experiences of a dietetic consultation have the potential to contribute to self-confidence with managing and transitioning to lifelong dietary changes. In order to achieve this, more research is needed to provide greater insight into the perspectives of individuals with coeliac disease and inform how dietitians can best assist in improving the lives of those living with coeliac disease in rural Australia.

## Figures and Tables

**Table 1:** Participant demographic information, symptoms at diagnosis, number of dietetic consultations, and other descriptive information.

Participant. MMM Location	Gender Age (Years)	Years since Diagnosis at Time of Interview	Length of Symptoms Prior to Diagnosis	Symptoms at Diagnosis	Number of Dietetic Consultations since Diagnosis	Household Description	Other Descriptive Information
P1 MM3	Female 38	3 ½	2 years	Diarrhoea, fatigue, ‘cognitive fogginess’, vitamin D and iron deficiencies	1	Living with husband and young children	Finds family life and socialising difficult within the constraints of the GF diet and often gives priority to the dietary needs of her family over her own.
P2 MM5	Male 69	3	20–30 years	Abdominal pain and distension	2	Living alone	Spends a considerable amount of time outside of the home due to travelling occupation, relying heavily on takeaway food.
P3 MM3	Male 77	2	N/A	No symptoms reported. Diagnosis as a result of routine gastroscopy	2	Living with wife	Very dependent on wife for support, dietary knowledge and meal provision,
P4 MM3	Male 66	≤1	1 year	Constipation, wind, abdominal pain	1	Living alone	Performs all cooking and shopping duties autonomously.
P5 MM5	Female 72	10	unclear	Abdominal symptoms and reflux	1	Living with husband	Lives in small rural town, distant from a major regional centre. Finds accessing GF food difficult locally.
P6 MM3	Female 58	8	Not specified	Fatigue, explosive loose bowel motions, anaemia, bloating and abdominal discomfort	5	Living with husband	Cooks at home, does not eat out often, finds food access reasonable. Husband chooses to eat GF at home.

GF—gluten-free, MM—Modified Monash classification.

**Table 2:** Themes, sub-themes and supportive quotes regarding perspectives of adults living with coeliac disease and accessing dietetic services in rural Australia.

Themes and Sub Themes	Supportive Quotes
**3.1 Optimising individualised support and services**	
3.1.1 Provision of support and services relevant to patient needs	‘They were able to point me in the right direction as to what foods are suitable for a gluten-free diet. So that was helpful… they carried out their service in a very informative and professional manner.’ (P6) ‘I think it [the dietetic consult] was a two-way process. They’re teaching me about things but they’re also good in as much as that they ask questions and they take on board the information that you give them. So I think it’s a repository for information backwards and forwards. That’s been quite worthwhile.’ (P3) ‘I was pleasantly surprised. I got attention and good advice. I think it was reinforced that I was actually on the right track. I was pretty happy with my experience.’ (P4) ‘We’ve gone into detail on my diet and how this could be happening and whether there’s any cross-contamination and things like that. So I can’t really say that I’ve been unhappy or unsatisfied with the service I’ve got from the dietitian.’ (P6) ‘It was just really basic information that everyday people know about… but you could sort of see I guess by looking at me and by the way I spoke that I didn’t need to be educated on basic healthy eating.’ (P1) ‘She didn’t give me all that much information. I mainly got it all out of the books.’ (P5) and that access to a dietitian was difficult due to the lack of a local service ‘a dietitian only comes here if you want to see one… at least once a month… if I wanted to see one I’d have to go to [regional centre] (P5).
3.1.2 Meeting the expectations of the patient	‘There’s some lipsticks you shouldn’t use, some shampoos and things like that that can affect your coeliac… there’s medication that has gluten in it… I would expect my dietitian to point [that] out to me’ (P1). ‘…having the support and learning the skills and getting the tools to do that, which I feel the dietitians here where I live have done that… they gave me all the tools and skills to go out into the big wide world and hit the supermarket aisles.’ (P6)
3.1.3 Consistency in communication and coordination of care	‘I think there needs to be a lot more conversing, communication between the doctors and the dietitians… at the beginning there… there just seemed to be no communication or something... he said, “Your dietitian will tell you. There’s lots of things that you don’t realise that have gluten”… I don’t think that he realised that I wasn’t actually told all that sort of stuff’. (P1) ‘I guess in some ways you don’t want someone to be sympathetic to you, but they are also understanding that it’s challenging and helping you to move forward with this new diet that you need to follow, and pointing out… as the GP did as well, like we all have choices.’ (P6)
3.1.4 Improving services and resources	‘If there was someone that… says they’re an expert in gluten-free cooking, we’d love to be aware of that’ (P3). ‘…the Coeliac Society has a good website and there’s another book… on gluten-free cooking… one that I use a lot.’ (P6)
**3.2 Adapting to a gluten-free diet in a rural context**	
3.2.1 Confidence around managing ones’ coeliac disease	‘I’m getting better at it [managing the gluten-free diet], definitely, but I’m still learning the whole time.’ (P2) ‘There’s a lot of food that is actually gluten-free but they don’t state it. I’m thinking to myself I should get that… Oh I better not because it doesn’t have the ‘gluten-free’ [label] on it.’ (P1) ‘You miss out on the pleasure of tasting something simply because you don’t enquire enough or recognise that it is within the bounds of what you can and can’t consume.’ (P3)
3.2.2 Adapting to and maintenance of the gluten-free diet	‘I’m certainly better and I feel better…’ (P4) and ‘I feel a lot of those symptoms have gone…’ (P6) ‘There may be the very odd occasion that I might have some gluten in my diet purely because I might be in a social situation… I just the pay the price a little bit later after eating gluten, if you know what I mean. So maybe in a social situation I might have a little bit of gluten very, very occasionally.’ (P6) ‘If it’s something new I’ve got to read the label… I’ve never read a label before, now I’ve got to.’ (P2) ‘It shouldn’t be that hard I don’t think… It should only take six months really.’ (P1) ‘Trouble is, if I do get an episode where I’ve got something [gluten] slipping through… It seems to take a little while before the symptoms disappear.’ (P2) ‘The cost is usually double... Everything’s a lot more expensive.’ (P2) ‘I cook myself, but I don’t bake that much. So I buy the bread, and that’s $6.99 [AUS$] a loaf… and you can only get about a dozen slices… I think it’s too expensive. I really do and especially in country areas where we are.’ (P5) ‘I struggle with the price because everything that seems to be gluten-free, such as a loaf of bread for instance is three times the price of a normal loaf of bread.’ (P6) ‘I’m not a high income earner… I had to take [that] into consideration and to learn how to shop within my budget.’ (P6) ‘I miss a good meat pie… and bread. I get a second-rate meat pie and I get the worst bread.’ (P2)
3.2.3 Food as a major aspect of life	‘I find eating out is just awful, I dread it sometimes.’ (P1) ‘Eating is one of those things that you either do automatically or you do because you like it and I don’t know that I’ve ever been one that ate automatically... So I don’t think I’ll ever forget the sensation of tasting something nice. So when you do eat something and you think to yourself, oh, that wasn’t much of a thrill, was it?’ (P3) ‘Everything is gluten-free’ [at home] but when out ‘I cannot follow it when I’m out, if I have somewhere to eat. You know what I mean?’ (P5) ‘Well to be quite honest, I haven’t really found it all that difficult because I guess I eat a pretty basic sort of diet and so there are some gluten-free products that I really don’t like and I just don’t eat but… I look at alternatives.’ (P6) ‘I found it difficult initially because a lot of the products had extra sugar and other components in there, so I was then struggling with the weight thing because of that. So instead of having some of these more processed foods… I look at more natural foods.’ (P6) ‘It’s very hard when you’re living in the country with only 1200 people here and you have to go out of town and… I can’t afford to keep going out of town to get the food… in the supermarket it’s good but you don’t get an awful lot of gluten-free. It’s mainly breakfast you can’t get… I cannot get the stuff I want to eat and what they’ve got, I don’t like.’ (P5) ‘My husband and I have travelled around Australia over the last few years so that was more challenging going into some pretty remote places where there was very little gluten-free products and if they were, you paid an enormous amount of money for them. So I guess again it was challenging because your fresh fruit and veggies that you would have normally were almost—even that was difficult to get hold of because of the availability and the price but you’d work around it.’ (P6)
3.2.4 Resignation to the diet and lifestyle changes	‘I’m still waiting for someone to ring me up and say, “Okay [refers to self], you’re right to go back to eating whatever you like” and sometimes I get this smack in the face and I just go, “Oh my god, that’s never going to happen. This is forever.” I’m never actually going to be able to just go and have fish and chips freely when I go on holidays again.’ (P1) ‘It’s one of these things. It’s like getting your leg cut off, there’s not much point wishing it hadn’t happened.’ (P2) ‘Your life doesn’t need to stop [when you have coeliac disease]; there are always ways around accommodating your likes and dislikes.’ (P3) ‘I just accept it. It’s no different than being a diabetic. It’s just what I need to do and I just do it.’ (P6) ‘I do enjoy baking, so that’s probably been the biggest hurdle… is learning how to bake with the gluten-free flour and different flours on offer. But in time you manage it and you get accustomed to it and you can get some pretty good results.’ (P6)
**3.3 Managing coeliac disease within the context of interpersonal relationships**	
3.3.1 The role of others	‘A friend of mine invited all her friends and me over for dinner the other night and I just felt like the biggest pain in the neck. I always have to just say, “Is it gluten-free, what you’re cooking” and they all go, “Oh, I forgot.” I just feel like a burden all the time and that sort of gets you down.’ (P1) ‘Socially, it’s a little bit embarrassing for the people mainly because if they put [food out] and [I] say, “Sorry I can’t eat it because I’m gluten-free”… I tend not to go out much anyway.’ (P2) ‘Other family members that invite you over and they sit you down and you’ve got a gluten meal in front of you and you think why don’t you get it? Why don’t you understand I cannot have gluten? ... If I don’t eat that, I go hungry and I don’t particularly want to say well I’ll come as long as you cook me a gluten-free meal because that then—it just becomes too hard for them and I don’t want to—I don’t want that to be the case.’ (P6) ‘I’m lucky that I’ve been associated with these people for probably 30 years so they say “Well what can we do [to help]?”….and they’re quite prepared to do that.’ (P3) ‘If it [the gluten-free diet] affects other people, too bad, because I’ve got to look after number one. That’s the way I look at it. And really, true friends will support that anyway.’ (P4) ‘…they certainly know that I’m coeliac and they came for morning tea the other day and had brought a cake which was from a supermarket which wasn’t gluten-free. So I cut it up and I didn’t have a piece… ‘Why aren’t you having a piece?’… And I don’t like to draw attention to myself. I don’t want to be a stick in the mud. And I’m going, ‘I’m coeliac, I can’t eat it.’… ‘Oh, I’m so sorry.’ (P6) ‘I’ve had café [staff] actually just like roll their eyes at me when I’ve said, “I need gluten-free” and it’s not until I’ve actually said I’m allergic to it.’ (P1) ‘It’s a more recognised complaint now and the supermarkets and whatever cater for it… the range has just expanded so much.’ (P3)
3.3.2 Management of the diet and disease within the family unit	‘I find it’s difficult when you’ve got little kids and I don’t know whether to go fully gluten-free in this house. For example, the butter dish, it’s always full of breadcrumbs, so I’ve gone down the track of doing two butter dishes. It’s really difficult. Do I have two butter dishes, two jam dishes, two honey? You know, it just gets a little bit out of control.’ (P1) ‘There’s no issues... if I was to go to my daughter’s houses or them to come here or vice versa there’s never any issue.’ (P6) ‘I know it’s my husband’s choice but at the time same time he pretty much has a gluten-free diet as well and doesn’t eat those sorts of foods in front of me.’ (P6)
3.3.3 Responsibility for the diet and disease	‘Other people don’t—which they don’t need to… take responsibility for what you put in your mouth… but that’s fine, I mean it’s hard enough sometimes me getting my head around it, let alone other people thinking about what I’m eating.’ (P1) ‘If I’m in an environment where I can control it… I can order something that’s gluten-free… whereas if you go into somebody’s home, it’s one thing to take some cheese and crackers. It’s another thing to take your own total meal. I wouldn’t feel comfortable doing that.’ (P6) ‘I understand and I appreciate their hospitality, but at the same token I don’t want sit up to two slices of white normal bread and then come home and be on the toilet all afternoon.’ (P6) ‘He [husband] brought home… A treat and I said “I assume you asked whether that’s gluten-free?” and he’s like “Um…”’ (P1). ‘I took my wife with me [to the dietitian]… she’s the one that looks at what’s on the shelf, reads the instructions, reads the contents and questions it... I’m lazy and she’s not, so she picks up the information and applies it.’(P3) ‘I’m blessed with a lovely wife who’s a thinking person and capable. I think if you had somebody that wasn’t capable of getting around these difficulties [coeliac disease management], it’d be a burden.’ (P3)

## Data Availability

The data presented in this study are available on request from the corresponding author. The data are not publicly available due to participant privacy concerns as included data has been appropriately de-identified.

## Supplementary Materials

The following are available online at https://www.mdpi.com/article/10.3390/nu13062074/s1, Semi-structured interview questions.
