# Supplementary material for: Patient Perspectives of Living with Coeliac Disease and Accessing Dietetic Services in Rural Australia: A Qualitative Study
Source: Nutrients. 2021 Jun 17;13(6):2074. doi: 10.3390/nu13062074 (PMC8234981; doi:10.3390/nu13062074)
Supplement: Supplementary file 1 [file nutrients-13-02074-s001.zip › nutrients-1185559-supplementary.pdf]

Supplementary File: Semi-structured interview questions

The following questions are in relation to the research study 'Retrospective evaluation of coeliac disease cases referred to a rural dietetics outpatient clinic' study

The following information is to be recorded as demographic data at the start of the interview.

Confirm and record verbal consent to participate in interview:

Yes / No, if No, do not continue interview with interview.

Gender: Male/ Female

Age:

How many years since coeliac disease diagnosis?

How many times have you seen a dietitian regarding the management of your coeliac disease?

Semi-structured interview questions on following page.

*Adapted from Dorn et al (2010). The development and validation of a new coeliac disease quality of life survey (CD-QOL). Alimentary Pharmacology and Therapeutics 31, 666-675.
